# Frost fighters: The discovery of a tripartite molecular module that confers cold stress tolerance in apple

**DOI:** 10.1093/plcell/koaf287

**Published:** 2025-12-19

**Authors:** Margot Raffeiner

**Affiliations:** Assistant Features Editor, The Plant Cell, American Society of Plant Biologists; Faculty of Biology and Biotechnology, Ruhr University Bochum, Bochum 44801, Germany

When exposed to freezing temperatures (below 0 °C) or chilling conditions (0 to 15 °C), it gets a little chaotic inside the cell. For example, proteins that usually keep cell membranes flexible and functional begin to malfunction, leading to injury and, in crop species such as apple (*Malus × domestica*), severe yield losses (Unterberger et al. 2018). To restore balance, plants rely on protein degradation pathways that help remove damaged or misfolded proteins. Two major systems take the lead: proteasomal degradation, where a large protease complex directly cleaves proteins, and autophagy, in which cargos are engulfed by vesicles called autophagosomes and delivered to the vacuole for enzymatic breakdown (Raffeiner et al. 2023).

Because cold stress is a serious problem in apple cultivation, considerable research has been directed toward uncovering the molecular mechanisms of cold tolerance. Recently, a key discovery was the identification of a negative regulator called MYB30-Interacting E3 Ligase 1 (MdMIEL1), a RING-type E3 ligase whose stability must be carefully controlled to allow normal development and proper stress responses (An et al. 2020).

In new work, Fang Zhi and colleagues (Zhi et al. 2025) dissected how MdMIEL1 is regulated under cold stress. First, they discovered that MdMIEL1 interacts with MdKIN10, the catalytic α-subunit of the plant master regulator kinase Sucrose nonfermenting 1 (SNF1)-related protein kinase 1 (SnRK1) (Shen et al. 2009). Interestingly, this study further revealed that MdKIN10 activity itself is increased specifically under cold conditions, highlighting its role in freezing tolerance. Not only does MdKIN10 interact with MdMIEL1, but it also phosphorylates it, marking it for degradation (Fig. 1).

**Figure 1.**
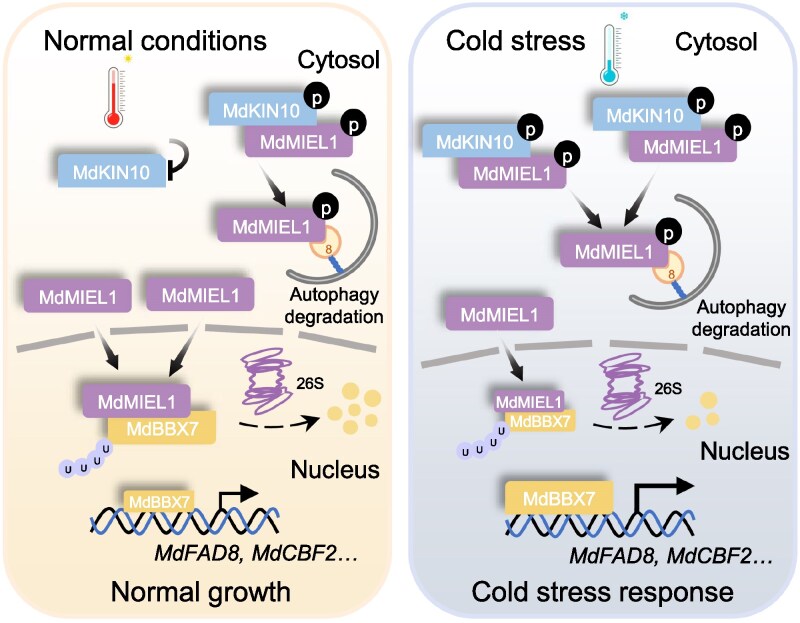
A tripartite molecular module confers cold stress tolerance in apple. The model illustrates that under normal growth conditions (left), MdMIEL1 is less phosphorylated by MdKIN10 due to reduced kinase activity. Therefore, MdMIEL1 escapes from its autophagic degradation and mediates ubiquitination and proteasomal degradation of its target protein MdBBX7, a positive regulator of cold stress response, ultimately reducing transcription of cold stress–responsive genes. In plants facing cold stress (right), MdKIN10 is highly active and promotes phosphorylation and subsequent autophagic degradation of MdMIEL1. In consequence, MdBBX7 is not degraded via the proteasome and can promote transcription of cold stress–responsive genes, boosting apple resilience toward cold temperatures. Adapted from Zhi et al. (2025), Figure 8.

To shed more light on the role of this interaction during cold acclimatization, the researchers generated transgenic apple calli that overexpressed either the wild-type (WT) MdMIEL1 or a nonphosphorylatable mutant (MdMIEL1^S198A^) alone or alongside MdKIN10. Under cold treatment, MdMIEL1 protein abundance decreased, but this effect was amplified by coexpression with MdKIN10. At the same time, increased phosphorylation of WT MdMIEL1 in MdKIN10 overexpressing calli was observed. In contrast, MdMIEL1^S198A^ protein remained stable, showing that phosphorylation by MdKIN10 is essential for MdMIEL1 turnover.

The changes in protein abundance of MdMIEL1 that depend on its phosphorylation status under cold stress were also reflected phenotypically. Plants overexpressing MdMIEL1 were more sensitive to cold, showing lower survival rates as well as higher electrolyte leakage, reflecting greater cellular damage. However, plants expressing a constitutively phosphorylated version (MdMIEL1^S198D^) showed better growth under cold stress compared with those expressing the nonphosphorylated version, further underscoring the relevance of MdMIEL1's phosphorylation for cold stress adaptation. The importance of KIN10 was also confirmed using transgenic plants: those overexpressing MdKIN10 had higher survival rates after freezing, whereas loss of MdKIN10 impaired tolerance.

But how is MdMIEL1 degraded? Testing different chemical inhibitors that block either autophagy or proteasomal degradation, the authors demonstrated that under cold stress, MdMIEL1 is cleared primarily via the autophagy pathway. Supporting this, MdMIEL1 was shown to interact directly with the Autophagy-related protein 8i (ATG8i), a key autophagy protein responsible for delivering substrates to autophagosomes. This interaction depended on MdMIEL1's phosphorylation status, again linking KIN10 activity to MdMIEL1 degradation. As expected, overall autophagic activity was higher during cold stress, helping the plant to eliminate this negative regulator when temperatures drop.

Ultimately, the study further found the protein MdBBX7 to be degraded via the proteasome after being targeted and ubiquitinated by MdMIEL1. MdBBX7 enhances cold tolerance by increasing cold response gene expression and promoting fatty acid desaturation to increase membrane fluidity. Together, the authors uncovered a regulatory network where MdKIN10 phosphorylates MdMIEL1 that is removed from the cell via autophagic degradation, thereby allowing positive regulators of cold stress response such as MdBBX7 to be activated.

In summary, this work identified a critical molecular module that helps apple trees to boost their tolerance to freezing temperatures, knowledge that might be applied in the future to bring more resilient apple varieties to market.

**Recent related articles in *The Plant Cell*:**

- Wang et al. (2023) identified a mechanism that fine-tunes the regulation of plant cold stress responses, where PUB25 and PUB26 E3 ligases regulate the stability of ICE1 and MYB15 by marking them with different ubiquitin chain types.
- Wang et al. (2025) discovered a high temperature–sensitive tripartite molecular module that inhibits the biosynthesis of anthocyanin, thereby likely being responsible for climate change-related fruit quality properties in pear.
- Zhang et al. (2025) showed that a noncanonical MAPK cascade that involves the kinases BSK4 and MAPK4 enhances cold tolerance in maize due to phosphorylation of bHLH transcription factors, which ultimately promote expression of cold-responsive genes.

## Data Availability

n.a.

## Dive Curated Terms

The following phenotypic, genotypic, and functional terms are of significance to the work described in this paper:

MYB15 Gramene: AT3G23250
MYB15 Araport: AT3G23250

PUB25 Gramene: AT3G19380
PUB25 Araport: AT3G19380

PUB26 Gramene: AT1G49780
PUB26 Araport: AT1G49780

ICE1 Gramene: AT3G26744
ICE1 Araport: AT3G26744

KIN10 Gramene: AT3G01090
KIN10 Araport: AT3G01090
